# Imaging Violence in Schizophrenia: A Systematic Review and Critical Discussion of the MRI Literature

**DOI:** 10.3389/fpsyt.2018.00333

**Published:** 2018-07-23

**Authors:** Maria Fjellvang, Linda Grøning, Unn K. Haukvik

**Affiliations:** ^1^Department of Mental Health and Addiction, Institute for Clinical Medicine, University of Oslo, Oslo, Norway; ^2^SIFER WEST, Haukeland University Hospital, Bergen, Norway; ^3^Faculty of Law, University of Bergen, Bergen, Norway; ^4^NORMENT K.G. Jebsen Centre for Psychosis Research, Oslo University Hospital, Oslo, Norway

**Keywords:** aggression, violence, amygdala, hippocampus, orbitofrontal cortex, anterior cingulate cortex, psychosis, forensic psychiatry

## Abstract

**Background:** Persons with schizophrenia have a small but significant increase in risk of violence, which remains after controlling for known environmental risk factors. *In vivo* MRI-studies may point toward the biological underpinnings of psychotic violence, and neuroimaging has increasingly been used in forensic and legal settings despite unclear relevance.

**Objectives:** (1) To present the first systematic review, following standardized guidelines, of MRI studies of violence with schizophrenia. (2) To critically discuss the promises and pitfalls of using this literature to understand violence in schizophrenia in clinical, forensic, and legal settings.

**Methods:** Following the PRISMA guidelines and literature searches until January 2018, we found 21 original studies that fulfilled the inclusion criteria: (1) Studies of persons with schizophrenia, (2) a history of violence or aggressive behavior, (3) the use of one or more MRI-modalities (sMRI, DTI, fMRI).

**Results:** The most consistent findings from the structural studies were reduced volumes of the hippocampus and the frontal lobe (in particular the orbitofrontal and anterior cingulate cortex) in schizophrenia patients with a history of violence or higher aggression scores. The functional studies mainly showed differences and aggression correlates in the frontal lobe and amygdala. However, the studies were methodologically heterogeneous, with four particular areas of concern: different definitions of violence, region of interest vs. whole-brain studies, small subject samples, and group comparisons in a heterogeneous diagnostic category (schizophrenia).

**Conclusion:** The literature reports subtle, but inconsistent group level differences in brain structure and function associated with violence and aggression with schizophrenia, in particular in areas involved in the formation of psychosis symptoms and affective regulation. Due to methodological challenges the results should be interpreted with caution. In order to come closer to the neurobiological underpinnings of violence in schizophrenia future studies could: (1) address the neurobiological differences of premeditated and reactive violence, (2) use RDoC criteria, for example, or other symptom-based systems to categorize psychosis patients, (3) increase subject cohorts and apply new data driven methods. In this perspective, MRI-studies of violence in schizophrenia have the potential to inform clinical violence prediction and legal evaluations *in the future*.

## Background

Persons with schizophrenia have a small but significant increased risk of violence (1). Although most persons with schizophrenia never commit a violent act, this association may cause fear and contribute to stigma toward a large group of patients. Violence is a multifactorial phenomenon that correlates with environmental factors such as substance abuse, low socio-economic status, and childhood trauma, which are also risk factors for schizophrenia (2, 3). However, the increased risk of violence remains after controlling for environmental factors (1, 2) which suggests that neurobiological factors may also be of importance to the increased risk of violence with schizophrenia.

Violence is a complex construct that may be conceptually separated into two categories, i.e., instrumental (planned, without activation of the autonomic nervous system) and impulsive (fear/aggression, with autonomic activation), which involve different neurobiological mechanisms (4). These categories do not, however, have strict boundaries, and both forms may occur simultaneously. Psychotic violence may, include both impulsive (disrupted affective regulation, misinterpretation of a threat based on delusions or hallucinations) and instrumental (based on delusions, hallucinations, personality traits or negative symptoms) aspects.

Sophisticated magnetic resonance imaging (MRI) techniques [anatomical (sMRI), diffusion tensor imaging (DTI), and functional MRI functional MRI (fMRI)] have allowed non-invasive studies of the living brain. MRI studies in forensic and clinical samples have confirmed that instrumental and impulsive violence are linked to alterations in specific brain circuits and areas, such as the amygdala, prefrontal cortex and the uncinate fasciculus which connects the two (4–7). Persons with psychosis show brain abnormalities, including smaller hippocampal volumes, widespread prefrontal and temporal cortical thinning (7–9), disrupted white matter integrity (10, 11), and altered functional connectivity (12). Such brain abnormities have been associated with clinical presentations; for example, hallucinations have been related to specific abnormalities in cortical morphology (sMRI) (13), white matter (DTI) (14), and abnormal functional connectivity (fMRI) (15).

Advances in neuroimaging methodology have led to an increased use of MRI-images in forensic and legal settings (16, 17). Such a use may be a valuable contribution to risk evaluations and as mitigating evidence but comes with critical concerns (17–19). The potential non-scientific use of neuroimaging data in forensic samples implies the need for a careful evaluation of their validity.

In this systematic review we ask: What is known about the structural and functional brain abnormalities of schizophrenia patients with a history of violence or aggression? How should the findings be interpreted with regard to underlying neurobiological mechanisms? And how can they impact on clinical, forensic, and legal evaluations?

Our first *aim* is to systematically review the literature by following the standardized PRISMA guidelines. Our second *aim* is to carefully and critically discuss the promises and pitfalls of using neuroimaging to study violence with schizophrenia, and how this literature may inform clinical, forensic and legal evaluations. We will point toward methodological challenges the field needs to overcome (the pitfalls) and present some recommendations for futures studies based on the literature reviewed, other emerging trends in imaging, and mental health research (the promises).

## Methods

**Figure 1:**
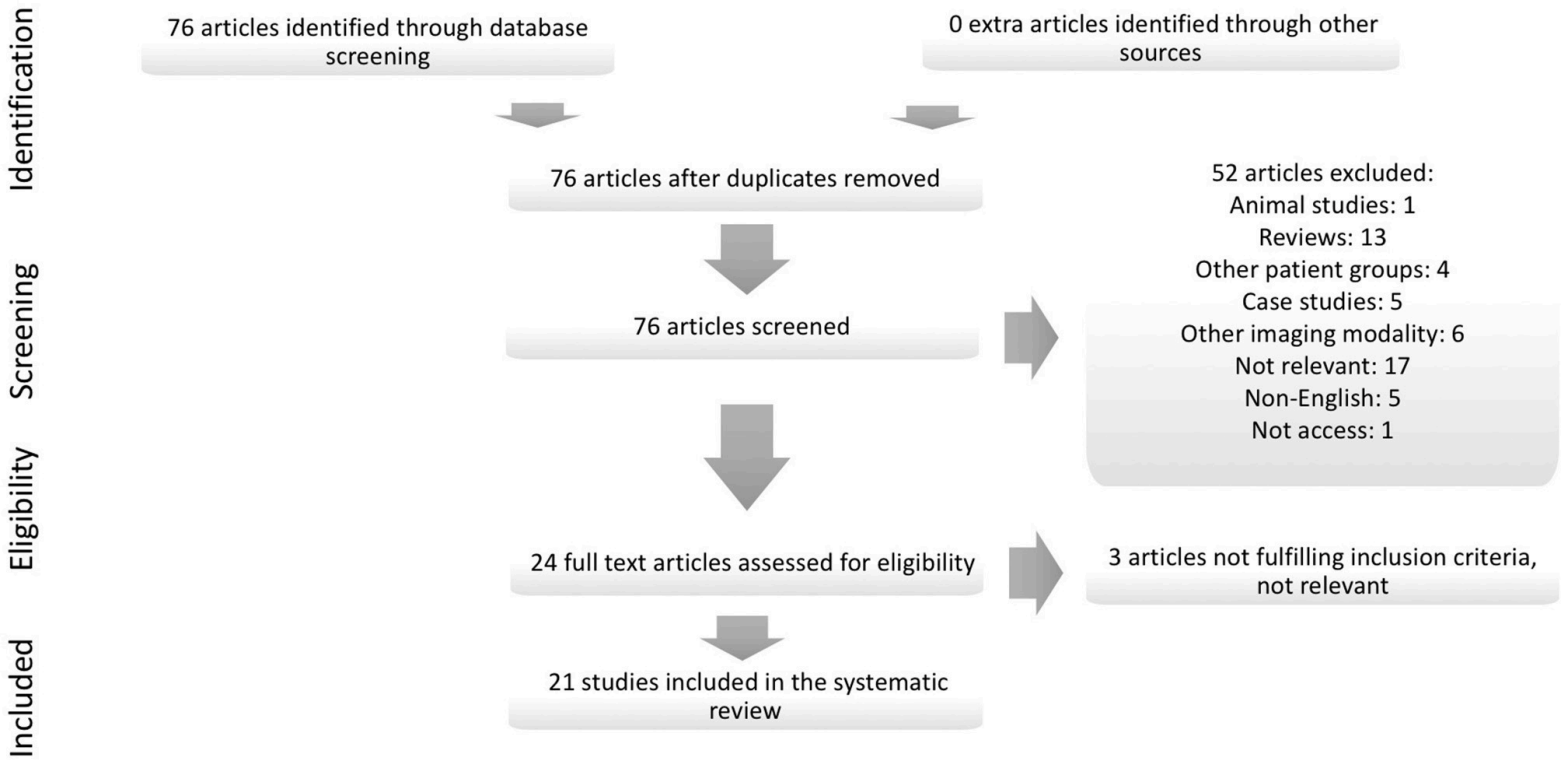
Detailed overview of the systematic article selection.

The systematic review is based on the Preferred reporting items for systematic reviews and meta-analyses (PRISMA) 27 item checklist and flow diagram (20). The literature search was performed in January 2018 in PubMed with the search phrase “schizophrenia AND (violence OR aggression) AND (MRI OR neuroimaging),” with no time limit. All abstracts were read for screening, and the eligibility criteria were: (1) original studies in English, (2) using MRI (including sMRI, DTI, or fMRI), (3) to assess violence or aggression, (4) in patients with schizophrenia. Post mortem-, animal-, and studies conducted on other patient groups were excluded, as were reviews. References were cross-checked for relevant studies. The screening and selection procedure is detailed in Figure 1.

## Results

The literature search returned 76 articles. All abstracts were read for screening. In total, 21 studies (21–41) fulfilled the eligibility and inclusion criteria. No extra studies that fulfilled the inclusion criteria were found by cross-checking references or cross-referencing in the search database. Of the 21 studies included in the systematic review, 14 studied violence as a categorical domain, and 7 studies of various measures of aggression, including impulsivity and psychopathy. Regarding MRI modality, there were 11 sMRI-, 1 DTI-, and 9 fMRI studies. The subject sample ranges were from 10 to 37 participants with schizophrenia and a history of violence when a categorical definition of violence was used; the smallest study using a dimensional measure of violence or aggression included 14 participants with schizophrenia (28). The largest study, independent of how violence was defined, included 111 participants across diagnostic categories (23). The main characteristics and findings from each study are summarized in Table 1.

**Table 1:** Structural MRI (sMRI, DTI) studies.

**Authors**	**Subject sample**	**Violence definition**	**ROI**	**Results**
**STRUCTURAL MRI**				
Barkataki et al. (22)^*^	vSCZ = 13 SCZ = 15 AP = 13 HC = 15	Gunn-Robertson Scale	Total brain volume, cerebellum, temporal lobe, ventricles, caudatus, putamen, thalamus, hippocampus and amygdala	vSCZ vs. SCZ reduced total brain volume reduced hippocampus and amygdala volume increased putamen volume
Del Bene et al. (23)	vSCZ = 37 SCZ = 26 VC = 24 HC = 24	Life history of aggression, criminal records	Subcortical structures	No specific volumetric abnormalities in vSCZ vs. SCZ
Hoptman et al. (25)	SCZ = 33 HC = 31	Urgency scale + Buss Perry Aggression Questionnaire	Frontal cortex	Higher impulsivity scores correlate with reduced cortical thickness in the orbitofrontal and anterior cingulate cortex in SCZ with aggression
Hoptman et al. (27)^#^	SCZ + SCA = 49	Overt Aggression Scale + PANSS	Nucleus caudatus	Higher aggression scores correlate with larger caudatus volume
Hoptman et al. (29)^#^	SCZ + SCA = 49	Overt Aggression Scale + PANSS	Orbitofrontal cortex	Increased gray and white matter volume in the orbitofrontal cortex (OFC), correlate with higher aggression levels
Kumari et al. (34)^*^	vSCZ = 13 SCZ = 15 AP = 14 HC = 15	Gunn-Robertson Scale	Anterior cingulate cortex	vSCZ vs. HC: lower anterior cingulate volume
Kumari et al., (32)	vSCZ = 10 SCZ = 14 FC = 14	Measure of impulsivity, history of severe violence	Prefrontal cortex, hippocampus and amygdala	Dysfunctional violence correlated with violence and reduced hippocampal and orbitofrontal cortex volume
Kuroki et al. (35)	vSCZ = 34 SCZ = 23	Murder, attempted murder, severe violence	Voxel-wise whole brain	vSCZ vs. SCZ lower gray matter volume in inferior and middle temporal gyrus, temporal pole, fusiform gyrus, insula and ventral diencephalon.
Narayan et al. (36)^*^	vSCZ = 12 SCZ = 15 AP = 14 HC = 15	Gunn-Robertson Scale	Across the entire cortex	vSCZ vs. SCZ: reduced cortical thickness in sensorimotor regions
Puri et al. (37)	vSCZ = 13 SCZ = 13	Subjectively evaluated by psychiatrist	Whole brain gray matter	vSCZ vs. SCZ reduced gray matter bilaterally in the cerebellum and BA 39/40
Schiffer et al. (38)	SCZ + CD = 27 SCZ = 23 HC = 25	Life history of aggression questionnaire	Whole brain gray matter	SCZ +CD vs. SCZ: reduced gray matter volume in the hypothalamus, putamen, left cuneus/precuneus and parietal cortex
Yang et al. (41)	vSCZ = 22 SCZ = 19 VC = 18 FC = 33	Murder	Prefrontal and limbic structures	vSCZ vs. SCZ, HC, VC: Reduced hippocampal gray matter bilaterally
**DTI**				
Hoptman et al. (28)	SCZ = 14	Buss Durkee Hostility Inventory + aggression history	Frontal-region	Reduced FA in the inferior frontal region correlate with higher impulsivity scores

*DTI, diffusion tensor imaging; ROI, region of interest; vSCZ, patients with schizophrenia and violence; SCZ, patients with schizophrenia; CD, patients with conduct disorder; VC, control persons with a history of violence; AP, patients with antisocial personality disorder; HC, healthy controls; PANSS, positive and negative syndrome scale; BA, Brodmann area/region; SCA, patients with schizoaffective disorder; FA, fractional anisotropy*.

* or # *same cohort*.

In the following, we will summarize the findings from the structural MRI-studies under the *Brain anatomy* heading, and the fMRI studies under the *Brain function* heading.

### Brain anatomy

Structural MR images provide a detailed anatomical overview. By the use of advanced post-processing methods, images from several persons are standardized according to a predefined atlas, which facilitates automated estimates of brain structure volumes, cortical morphology, and gray to white matter ratios. As such, brain anatomy across different clinical groups and phenomena may be quantitatively compared.

The most severe form of violence is homicide. Yang and colleagues found smaller hippocampal volumes in schizophrenia patients who had committed murder compared to schizophrenia patients without a history of violence, murderers without schizophrenia, or healthy controls (41). Smaller hippocampal volumes were also reported in another study of schizophrenia patients with a history of severe violence (22); this study also found reduced amygdala and total brain volume. In an overlapping study of the same patients, Kumari and colleagues found that the patients with a history of violence had increased dysfunctional or aggressive impulsivity which correlated with reduced hippocampal- and orbitofrontal cortex (OFC) volume (32). In contrast, in the hitherto largest study comprising 37 schizophrenia patients with a history of violence, the authors report no significant differences in hippocampus or amygdala volume compared to non-violent schizophrenia patients (23). The hippocampus is involved in learning, memory and the cognitive processes of pattern completion and pattern separation (42). Disturbances in this ability may contribute to the formation of delusions and hallucinations (42), which are psychosis symptoms related to violence.

The findings of reduced OFC volume concur with the results of a study of schizophrenia patients in which Hoptman and colleagues found increased impulsivity to correlate with cortical thinning in the OFC and anterior cingulate cortex (ACC) (25), and to correlate with aggression, which led the authors to suggest that aggression could also be correlated with thinning of the OFC. Kumari and colleagues later reported reduced volume of the ACC in schizophrenia patients with a history of violence, but only compared to healthy controls (not to non-violent schizophrenia patients) (34). The ACC is part of the limbic system and is, together with the amygdala, important to affective regulation. The OFC has earlier, also together with the amygdala, been associated with psychopathic traits (4). In contrast to the findings of reduced OFC-volume and OFC-thinning, another study by Hoptman and colleagues found higher aggression scores among schizophrenia patients to correlate with larger gray and white matter volumes of the OFC (29). In this latter cohort, the higher aggression scores also correlated with increased volume of the nucleus caudatus in the basal ganglia (27). The dopamine-fiber-rich basal ganglia volumes are particularly affected by the use of antipsychotics (43). Specifically, first-generation antipsychotics have been associated with enlargements of striatal volumes, and these effects may be particularly relevant for treatment-resistant patients who have undergone long term ineffective treatment with first-generation antipsychotics (44, 45). As such, this finding is somewhat difficult to interpret.

All the studies described hitherto were hypothesis driven regarding which brain regions were analyzed; they were so called region-of-interest (ROI) studies. In contrast, exploratory studies analyze the whole brain or cortex. An exploratory study by Narayan and colleagues found cortical thinning in sensory-motor areas of schizophrenia patients with a history of violence compared to non-violent schizophrenia patients (36). However, the cortical thickness did not differ between the groups in the OFC or ACC as may have been expected based on the findings reported above. By studying the whole brain gray matter of schizophrenia patients with a history of murder, attempted murder or severe violence, with larger subject sample, Kuroki and colleagues found reduced temporal, fusiform and insular volumes compared to non-violent schizophrenia patients (35). Patients with predatory violence showed more widespread volume reductions than patients with impulsive violence, but the OFC and ACC were not significantly reduced in either group. Puri and colleagues compared gray matter across the whole brain between violent and non-violent schizophrenia patients and found reduced volumes of the cerebellum and the parietal lobe, more specifically in regions near Wernickes area important to language formation (37). Schiffer and colleagues also studied the whole brain gray matter, but with a different definition of violence than Puri; they found that patients who had been diagnosed with conduct disorder prior to having been diagnosed with schizophrenia had a reduced volume of the hypothalamus, putamen (basal ganglia), and cuneus and parietal cortex compared to schizophrenia patients without conduct disorder (38). Neither of the two latter exploratory studies found differences in the regions reported in the hypothesis driven studies, and their results were also not overlapping.

A different method by which brain anatomy can be studied is diffusion tensor imaging (DTI). This method uses the diffusion properties of water molecules to provided different measures of white matter integrity and structural connectivity. One of the measures is fractional anisotropy (FA) which reflects the direction of the water molecules and the integrity of white matter tracts. In the only DTI-study to date (ROI based), Hoptman and colleagues found a correlation between reduced FA in the frontal cortex and increased measures of impulsivity, which correlated with aggression (28). This suggests that reduced prefrontal structural connectivity may be of importance for aggression in schizophrenia.

For a detailed overview of the methods and findings for each of the structural studies, please see Table 1.

### Brain function

The fMRI studies use measure of the blood oxygenation level (the BOLD response) in the brain while the test subject performs a task (task-based fMRI) or is at rest (resting state fMRI). Higher oxygen usage is considered a proxy for neuronal activity, and the method is used to map which parts of the brain that are active during a given task or in rest, that is as a measure of brain function.

Two hypothesis-driven fMRI-studies explored comorbidity and violence in schizophrenia. Joyal and colleagues (30) studied go/no go response in the frontal regions of murderers with schizophrenia with and without concurrent substance abuse and antisocial personality disorder. They report *reduced* activation in or near the OFC in the murderers with schizophrenia (and no comorbidity), whereas those with schizophrenia and comorbidity had *increased* activation in Broca's area compared to healthy controls. Dolan and colleagues studied amygdala activation in schizophrenia patients convicted for violence. They found patients with high psychopathy scores to have lower activation in response to fearful faces and higher activation in response to faces showing disgust compared to patients with low psychopathy scores (24).

Five other task-based fMRI-studies explored the whole-brain. Kumari and colleagues used an *affective expected fear paradigm* and found increased activity in the medial frontal and cingulate gyri and temporal-occipital regions as well as increased thalamo-striatal activity in schizophrenia patients with a history of violence (33). Another study of the same subject cohort showed an association between a history of violence and lower activity in the schizophrenia patients' right inferior parietal region during *the working memory n-back paradigm* (31). The third study from this cohort showed no differences between schizophrenia patients with and without a history of violence on *the adverse response/impulse inhibition go/no go paradigm* (21). In a different cohort, Tikaz and colleagues used an *affective go/no go paradigm* and found lower activation in the dorsolateral prefrontal cortex for angry faces in schizophrenia patients with a history of severe violence compared to non-violent schizophrenia patients. They link the findings to cognitive control and anger processing (40). In a separate study of an overlapping cohort, the authors used a *facial affect paradigm*. They found higher activation in the anterior cingulate, lingual, and precentral cortices in response to negative faces, and higher activation in the occipital, fusiform, and lingual cortices in response to neutral faces in the schizophrenia patients with a history of violence (39). Taken together, the results suggest a different pattern of activation during fear and complex tasks in schizophrenia patients with a history of violence. These abnormalities seem to be related to psychopathic traits, and they are suggestive of differences in cognitive control related to anger.

While task-based studies show brain function during externally initiated cognitive processes, the resting-state studies show regions that are active and communicate when the brain is resting or processing internally generated stimuli [the functional connectivity (FC)]. Hoptman and colleagues found a correlation between higher aggression scores in schizophrenia patients and lower FC between the amygdala and ventral prefrontal cortex in a hypothesis-driven study with the amygdala as the seed (26). In another study, the authors explored FC within the frontal cortex in schizophrenia patients and found an association between lower FC between the OFC and several regions, including the ACC, and negative impulsivity, and the negative impulsivity scores correlated with aggression scores (25). The results support the relevance of the ACC and OFC in violence and aggression in schizophrenia and they are in concordance with studies of non-psychotic violent offenders (46–48).

For a detailed overview of the methods and findings for each of the functional studies, please see Table 2.

**Table 2:** Functional MRI (fMRI) studies.

**Authors**	**Sample size**	**Violence definition**	**Task**	**ROI**	**Findings**
**TASK-BASED fMRI**					
Barkataki et al. (21)^*^	vSCZ = 12 SCZ = 13 AP = 14 HC = 14	Gunn-Robertson Scale	Go-no go	Voxel-wise, whole brain	vSCZ vs. FK: lower activation in thalamus and caudate vSCZ vs. SCZ: no difference
Dolan and Fullam (24)	12/12 vSCZ with high/low PCL	Conviction for violence/Psychopath Checklist	Facial affects	Amygdala and prefrontal cortex	High PCL correlated with lower amygdala activation to fearful faces and higher activation to disgust
Joyal et al. (30)	vSCZ = 12 vSCZ + AP + SA = 12 HC = 12	Murder	Go-no go	BA 9,10,11,44, 45,46,47	vSCZ: lower activation in BA10,47 vSCZ+AP+RM: higher activation in BA10,44,45
Kumari et al., (33)^*^	vSCZ = 13 SCZ = 13 AP = 13 HC = 14	Gunn-Robertson Scale	Affective, expected fear	Voxel-wise, whole brain	vSCZ vs. SCZ: higher activity in medial frontal/cingulate + temoraloccipitale regions
Kumari et al. (31)^*^	vSCZ = 12 SCZ = 13 AP = 10 HC = 14	Gunn-Robertson Scale	n-back, working memory	Voxel-wise, whole brain	vSCZ vs. SCZ: lower activity in the inferior parietal region
Tikasz et al. (40)^#^	vSCZ = 24 SCZ = 23 HC = 22	Homicide or severe violence	Affective go/no-go	Voxel-wise, whole brain	vSCZ vs. SCZ lower activation in the dorsolateral prefrontal cortex for angry faces
Tikasz et al. (39)^#^	vSCZ = 20 SCZ = 19 HC = 21	Armed aggression resulting in injury to others or death	Facial affects	Voxel-wise, whole brain	vSCZ vs. SCZ/HC: higher activation in anterior cingulate-, lingual-, precentral cortex to negative and occipital-, fusiform-, lingual- cortex to neutral faces
**RESTING STATE fMRI**					
Hoptman et al. (25)	SCZ = 33 HC = 31	Urgency aggression-scale	Seed based	Inferior frontal-regions	Lower functional connectivity correlated with higher urgency which correlated with aggression
Hoptman et al. (26)	SCZ/SCA = 25 HC = 21	Buss Perry Aggression Questionnaire	Seed based	Amygdala	Lower FC between amygdala and ventral prefrontal cortex correlated with higher aggression scores

*ROI, region of interest; vSCZ, patients with schizophrenia and violence; SCZ, patients with schizophrenia; AP, patients with anti-social personality disorder; HC, healthy; PCL, Psychopath Checklist; SA, substance abuse; BA, Brodmann area/region; SCA, patients with schizoaffective disorder; FC, functional connectivity*.

* or # *Same/overlapping subject cohort*.

## Discussion

**Figure 2:**
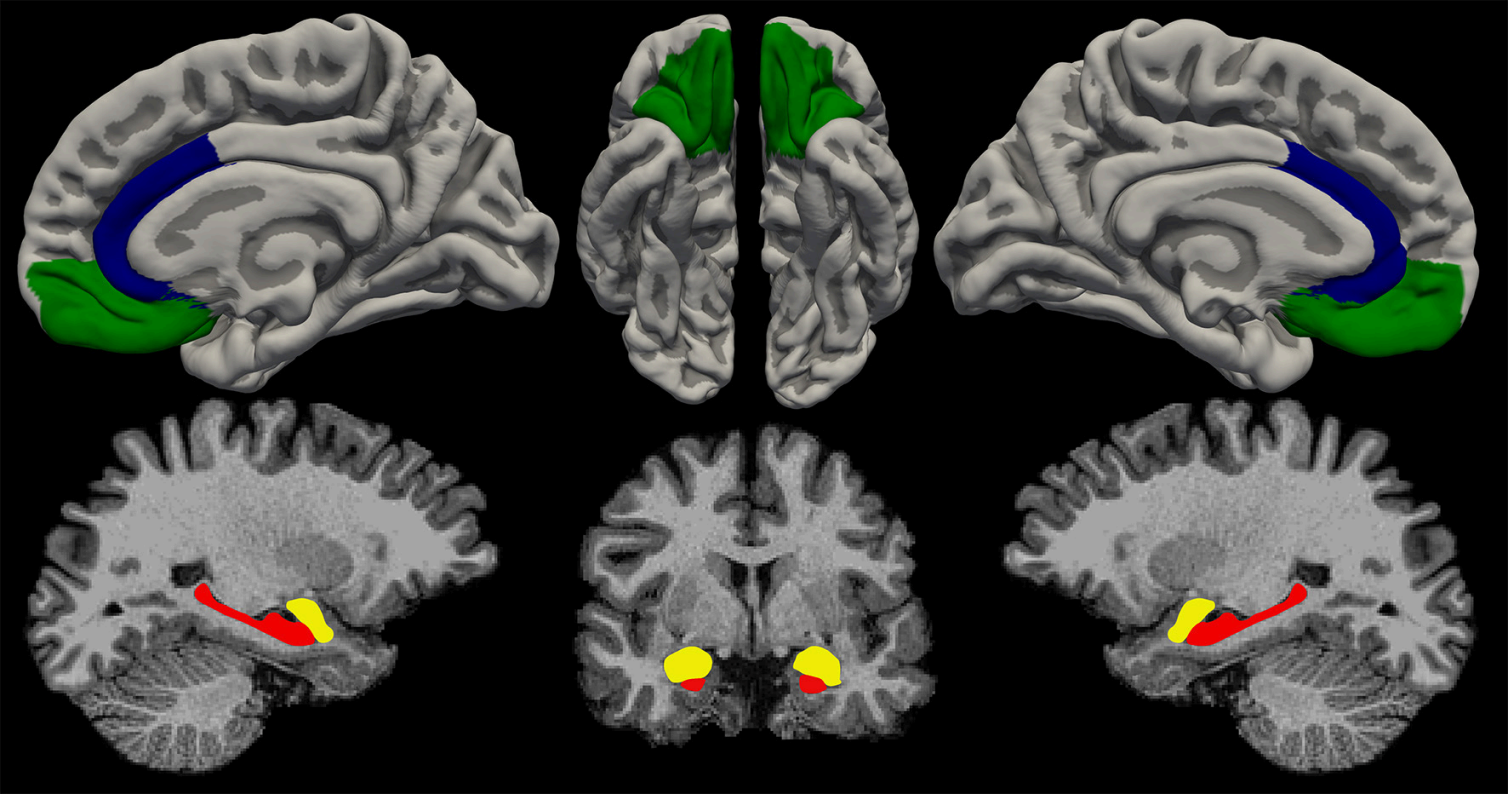
Illustration of the most replicated brain regions associated with violence and aggression. Red, hippocampus; yellow, amygdala; green, orbitofrontal cortex; blue, anterior cingulate cortex. Figure by: Stener Nerland.

A systematic review of the MRI literature suggests subtle brain structural and functional correlates of violence and aggression in schizophrenia, in particular in regions of importance to affective regulation (amygdala, OFC, ACC) and the formation of psychosis symptoms such as delusions and hallucinations (hippocampus, frontal cortex) (Figure 2). However, the mixed findings make a unifying interpretation of the results difficult and even controversial. Moreover, the reported brain-structure and connectivity abnormalities are not specific to violence in schizophrenia but are to some extent also present in other non-violent patient groups and non-psychotic violence cohorts. In the following, we will critically discuss the methodological challenges (the pitfalls) and present some recommendations for future studies (the promises).

*Challenge 1: The definition of violence* varies between studies. Some use a categorical definition such as murder or conviction for a violent act, whereas others use scales measuring violence related traits such as aggression and psychopathy. Four of the sMRI and two of the fMRI studies are based on aggression scores or impulsivity which include verbal threats, violence against objects, and self-injury. But even among the studies that used a categorical definition of violence (murder, severe violence, or conviction for violence) the results differ; they include reduced hippocampal volume, abnormal prefrontal and amygdala activation, and regional gray matter volume reduction in the hypothalamus, putamen, cuneus, and parietal cortex. As such, they prove difficult to integrate into a neurobiological model for violence in schizophrenia.

The characterization of violence according to models of impulsive or instrumental violence has not been addressed, except in one study (35), which reported more widespread cortical volume reductions in instrumental than in impulsive violence. Explicit information regarding the presence of psychosis symptoms (i.e., hallucinations, delusions, thought disturbances) and their manifestation during the violent act is lacking. Such symptom characteristics could be associated with specific brain-structure or function abnormalities involved in psychosis as well as violence and should be investigated with regard to possible interaction effects that could be relevant for a neurobiological model of violence in psychosis.

The time window between violent acts and scanning has not been reported. As such it is not clear if the reported group differences in brain structure or function represent *state or trait* characteristics of violence. The studies using impulsivity or aggression scales may better inform current psychopathology and state characteristics since this information collected at scanning time. However, one limitation with this approach is that the definition of violence may be too vague to capture the putative neurobiological mechanisms underpinning each domain. A recommendation for future neuroimaging studies on schizophrenia is to explore the distinct anatomical patterns of the brain the and functional correlates of instrumental and affective violence separately, and in addition clarify state vs. trait characteristics. Thereby, the field may come closer to mapping the specific neurobiological underpinnings of psychotic violence and clarifying whether psychotic violence represents a distinct category of violence in addition to instrumental and affective violence.

*Challenge 2: The analysis approach (i.e., hypothesis-driven vs. whole-brain studies)* differs between the studies. Hypothesis-driven (ROI) studies may be biased toward findings in brain regions that have previously been associated with violence in schizophrenia, and possible associations in other parts of the brain may be overlooked. In contrast, whole-brain studies avoid this problem, but may, through stringent adjustment for multiple comparisons, miss subtle differences. Indeed, the two explorative whole-cortex studies of thickness (36) or volume (35) did not confirm the OFC and ACC findings from the hypothesis-driven ROI-based studies. This lack of consistency between the studies causes concern. Given the limited MRI literature on violence and schizophrenia, the literature on non-psychotic violence may provide a framework for interpreting results and generating hypotheses. For instance, a recent study found increased functional connectivity between specific sub-regions of the amygdala and the prefrontal cortex in persons with psychopathic (callous-unemotional) traits (49). Another recent elegant DTI study on a large cohort of healthy subjects found an association between impulsivity and accumbo-frontal structural integrity; these results link the *in vivo* brain characteristics of impulsivity to animal models, where glutaminergic projections have been linked to impulsive behavior (50).

*Challenge 3*: Several of the studies included in this review were conducted on *small subject samples* (sometimes the cohorts of different papers overlap), comprising 10–15 subjects with schizophrenia and a history of violence. Worth noting is the fact that the largest study to date of subcortical volumes did not find significant reductions in amygdala or hippocampus volume in schizophrenia patients with a history of violence compared to those without (23). The neuroimaging field has acknowledged the risk of false positives in small subject samples and is studying increasingly larger subject samples in the search for subtle brain abnormalities associated with mental disorders (e.g., the ENIGMA consortia) (9, 51, 52). However, given the challenges with consent, safety, and severe psychopathology, small subject samples will continue to be an inherit limitation when conducting imaging-research in schizophrenia patients with a history of violence. To overcome this challenge, data from different cohort could be merged for a mega-analysis, while controlling for the different MRI-acquisition parameters and violence definitions.

*Challenge 4:* All the studies included in this review report on *group differences*. This is in line with most neuroimaging research in psychiatry which is based on characterizing differences in structure and function between diagnostic groups. The diagnostic group approach has been challenged by the recent emergence of research domain criteria (RDoC) in mental health research, which argue for studying the distinct neurobiological underpinnings of specific symptoms and phenomena (e.g., hallucinations, delusions) rather than diagnostic categories (53). Indeed, schizophrenia is a heterogeneous disorder comprising symptoms which are thought to have different neurobiological underpinnings (54, 55) This may problematize imaging studies and may blur the distinction between the neurobiology of the disorder and that of the different types of violence (as discussed under Challenge 1 above). As such, we recommend that future studies should seek to investigate how the specific symptom domains are related to the neurobiology of violence. By creating more homogenous groups, the putative associations to violence may become clearer and more similar between studies.

Bearing in mind the challenges and limitations discussed so far, are there any areas where neuroimaging violence in schizophrenia could be useful in clinical and forensic practice?

Recent advances in neuroimaging pattern classification methods and *prediction* tools have been promising with regard to single subject prediction (56), in particular if combined with clinical and behavioral data (57, 58). Of specific interest to the forensic use of neuroimaging, machine learning methods have been used for neuroimaging-based classification of psychopaths (59), to predict psychosis course (58, 60, 61), and culpability (62), as a proof of concept to show the potential of such methods. If neuroimaging methods could help to predict future violent behavior, this would impact the legal system with regard to sentencing, crime prevention and treatment (63). Indeed, despite the inherent difficulties regarding causality and single subject validity, MR images are used in the courts today, perhaps most notably as mitigating evidence in capital cases in the USA (16, 17). With regard to the potential legal use of MR images of schizophrenia patients, such use could be related to risk of violence evaluations, but also serve to inform the concepts of intention, insanity, criminal responsibility, and, ultimately, free-will (18, 19, 64). A recent elegant study of brain lesions in criminal offenders found a specific network of connectivity abnormalities between regions involved in moral decision making and theory of mind (46). In a sample of incarcerated youths, a data-driven MRI-study could predict the risk of violent re-offending after release (59). However, despite these promising studies, the neuroimaging field is still far from detecting single-subject biomarkers of violence prediction. Even if they were, a major challenge is that concepts such as free will, insanity, and morality are not similarly defined and understood in neuroscience and law. As such, the findings from the imaging literature, even if the studies were methodologically flawless, would not be directly transferable to legal practice (65). Moreover, other mechanisms such as abnormal serotonin metabolism (66) and immune marker profiles (67) may affect aggressive behavior, but their relationship to brain structure or connectivity in psychotic violence have not, to date, been clarified.

Finally, MR images are powerful visual tools. They may mislead non-scientists into believing there are significant, objective physiological correlates to violent behavior, when the images merely reflect statistical differences between groups that are *correlational and not causal* (the inferential distance) (17). Despite the possibility to overcome many of the challenges discussed above, forensic and legal practitioners need to be aware of this premise.

## Conclusion

This systematic review points toward subtle, but inconsistent, group-level differences in brain structure and function associated with violence and aggression in schizophrenia. Differences are reported in areas involved in the formation of psychosis symptoms and affective regulation, but are not coherent across studies. The findings suggest that neurobiological factors could be of importance to violence and aggression with schizophrenia, but due to methodological challenges, the results cannot, to date, be used in clinical or forensic evaluations. In order to come closer to the neurobiological underpinnings of violence in schizophrenia we recommend future research to: (1) deconstruct violence into the neurobiologically different premeditated and reactive categories, and specifically address state vs. trait measures of violence; (2) use RDoC criteria or other symptom-based systems to categorize psychosis patients rather than the broad and heterogeneous diagnostic criteria of schizophrenia; (3) increase subject cohorts (or combine cohorts with similar definitions of violence), and apply new data driven methods. In this perspective, MRI-studies of violence in schizophrenia have the potential to inform clinical violence prediction and legal evaluations *in the future*.

## Author contributions

MF performed the initial literature search, selected and read the articles, and wrote the first draft of the manuscript. LG reviewed and supervised the legal aspects of the study. UH performed the final literature search, selected and read the articles, supervised the first draft and wrote the final draft of the manuscript. All authors have contributed to and approved the final version of the manuscript.

### Conflict of interest statement

The authors declare that the research was conducted in the absence of any commercial or financial relationships that could be construed as a potential conflict of interest.
